# Keyhole limpet hemocyanin induces innate immunity via Syk and Erk phosphorylation

**DOI:** 10.17179/excli2016-488

**Published:** 2016-08-03

**Authors:** Kyoko Yasuda, Hideki Ushio

**Affiliations:** 1 Laboratory of Marine Biochemistry, Graduate School of Agricultural and Life Sciences, The University of Tokyo

**Keywords:** keyhole limpet hemocyanin, innate immunity, NF-kappaB, Syk, Erk

## Abstract

Hemocyanin is an extracellular respiratory protein containing copper in hemolymph of invertebrates, such as Mollusk and Arthropod. Keyhole limpet hemocyanin (KLH) is one of hemocyanins and has many years of experience for vaccine developments and immunological studies in mammals including human. However, the association between KLH and the immune systems, especially the innate immune systems, remains poorly understood. The aim of this study is to clarify the direct effects of KLH on the innate immune systems. KLH activated an inflammation-related transcription factor NF-κB as much as lipopolysaccharide (LPS) in a human monocytic leukemia THP-1 reporter cell line. We have found that the KLH-induced NF-κB activation is partially involved in a spleen tyrosine kinase (Syk) pathway. We have also successfully revealed that an extracellular signal-regulated kinase (Erk), a member of mitogen-activated protein kinases, is located in an upstream of NF-κB activation induced by KLH. Furthermore, a Syk phosphorylation inhibitor partially suppressed the Erk activation in KLH-stimulated THP-1. These results suggest that both Syk and Erk associate with the KLH-induced NF-κB activation in the human monocyte.

## Introduction

Hemocyanin is a large molecule and copper-containing protein and carries oxygen to peripheral tissues in mollusk and arthropod circulation (Amkraut et al., 1969[1]). Some of hemocyanins such as *Concholepas concholepas* hemocyanin (De Ioannes et al., 2004[12]; Moltedo et al., 2006[35]), *Fissurella latimarginata* hemocyanin (Arancibia et al., 2014[2]) and *Megathura crenulata* hemocyanin have been used as a hapten carrier to produce antibodies (Becker et al., 2014[4]). The marine mollusk *M. crenulata* hemocyanin is commonly known as a keyhole limpet hemocyanin (KLH). KLH is composed of two subunits of approximately 360,000 - 400,000 in monomeric molecular weight (Markl et al., 1991[31]) and well known as an immunostimulant in mammals including human for more than 40 years (Curtis et al., 1970[11], 1971[10]; Herscowitz et al., 1972[19]; Weigle, 1964[50]). However, the initial physiological responses to KLH and direct effects of KLH on the innate immune systems are not clear. 

The innate immune system plays an important role in host defense against many pathogens. Pathogen-associated molecular patterns (PAMPs) are detected through specific pattern recognition receptors (PRRs) (Janeway and Medzhitov, 2002[20]; Medzhitov and Janeway, 1997[32]; Mogensen, 2009[34]), which include toll like receptors (TLRs) and C-type lectin receptors (CLRs) (Drummond et al., 2011[14]). The important roles in host defense are production of inflammatory mediators and phagocytosis. For example, a gram-negative pathogen *Francisella tularensis* is recognized by PRRs, leading to the production of inflammatory mediators through the activation of mitogen-activated protein kinases (MAPK) and nuclear factor-kappa B (NF-κB) (Golovliov et al., 1996[17]; Stenmark et al., 1999[47]; Parsa et al., 2006[39]; Butchar et al., 2007[7]). The host cell receptors, including complement receptor 3 (CR3) (Ben Nasr et al., 2006[5]; Clemens et al., 2005[9]), mannose receptor (MR) (Balagopal et al., 2006[3]), toll like receptor 2 (TLR2) (Katz et al., 2006[22]; Malik et al., 2006[29]) and Fcγ receptors (FcγRs) (Balagopal et al., 2006[3]) are implicated in the recognition of *F. tularensis*. These receptors in myeloid cell mainly activate the downstream signaling pathways via spleen tyrosine kinase (Syk) (Osorio and Reis e Sousa, 2011[37]). Syk is one of the common molecules associated with these receptors (Kerrigan and Brown, 2010[23]; Hadas et al., 2012[18]; Dennehy et al., 2008[13]; Falker et al., 2014[15]). The study on *F. tularensis* phagocytosis also reported that Syk-dependent phagocytosis was controlled through an extracellular signal-regulated kinase (Erk) pathway (Parsa et al., 2008[38]). Erk is a member of the MAPK family and the MAPK cascades involve in the regulation of cell proliferation, survival and differentiation (Roberts and Der, 2007[42]). The MAPK pathways relay intracellular signals and elicit physiological responses such as inflammatory responses and apoptosis in mammalian cells (Roux and Blenis, 2004[44]; Zhang and Liu, 2002[52]). Several studies reported that Erk was a downstream component of many signaling pathways with various receptors, such as CR3 (Li et al., 2014[27]), MR (Tsai et al., 2013[48]), TLR2 (Richardson et al., 2015[41]; Chen et al., 2015[8]) and FcγRs (Luo et al., 2010[28]; Song et al., 2004[46]).

In this study, we have evaluated the effects of a Syk specific inhibitor on KLH-induced NF-κB activation and Erk activation in the human monocyte leukemia cell line THP-1. We then discussed about the roles of Syk and Erk in innate immune responses of THP-1 to KLH. 

## Materials and Methods

### Antibodies and reagents

Mariculture keyhole limpet hemocyanin was purchased from Thermo Fisher Scientific (Rockford, IL). Ammonium pyrrolidinedithiocarbamate (PDTC) and *Echerichia coli* LPS were purchased from Sigma-Aldrich (St. Louis, MO). Syk inhibitor (Bay 61-3606 hydrochloride), Erk inhibitor (Nimble) and anti-Erk1/2 antibody (EPR 17526) were purchased from Abcam (Tokyo). Anti-phospho-Erk1/2 (Tyr 202/204) antibody was purchased from Cell Signaling Technology (Danvers, MA). 

### Cell culture

THP-1 cells were obtained from Japanese Collection of Research Bioresources Cell Bank (Osaka) and grown in an RPMI medium (Thermo Fisher Scientific, Waltham, MA) supplemented with 10 % (vol/vol) fetal bovine serum (Thermo Fisher Scientific), 1 % (vol/vol) GlutaMAX™ Supplement (Thermo Fisher Scientific), and 100 μg/ml Normocin™ (InvivoGen, Sandiego, CA) at 37° C with 5 % CO_2_. THP1-Xblue™-MD2-CD14 cells were acquired from InvivoGen and grown in an RPMI medium supplemented with 10 % fetal bovine serum, 100 μg/ml Normocin™ (InvivoGen), 200 μg/ml Zeocin™ (InvivoGen) and 250 μg/ml G418 (InvivoGen) at 37° C with 5 % CO_2 _. THP1-Xblue™-MD2-CD14 cells contain the secreted embryonic alkaline phosphatase (SEAP) reporter gene under controls of NF-κB and/or activator protein-1 (AP-1).

### Measurement of NF-κB and/or AP-1 activity using THP1-XBlue™-MD2-CD14 cells

THP1-XBlue™-MD2-CD14 cells (5 × 10^5^ cells) were seeded at 96 well plate and then stimulated with 0.1 µg/ml LPS (positive control) or 50 µg/ml KLH for 24 h. Supernatants were collected and incubated with QUANTI-Blue™ (InvivoGen) for 24 h, which turns purple in the presence of secreted alkaline phosphatase (SEAP). SEAP levels were determined spectrophotometrically at 650 nm. 

### Western Blot Analyses

THP-1 cells (1 × 10^7^ cells) were stimulated with 0.1 µg/ml LPS or 500 µg/ml KLH at 37° C for various periods. At the end of the incubation period, cells were lysed in a RIPA buffer (20 mM Tris-HCl [pH 7.5], 150 mM NaCl, 1 mM disodium ethylene-diaminotetraacetate [Na_2_EDTA], 1 mM ethylene glycol tetraacetic acid [EGTA], 1 % Nonidet P-40 [NP-40], 1 % sodium pyrophosphate, 1 mM β-glycerophosphate, 1 mM Na_3_VO_4 _, 1 µg/ml leupeptin, Protease Inhibitor Cocktail [Nakarai Tesque, Tokyo], 1 mM NaF and 1 mM phenylmethylsulfonyl fluoride [PMSF]) for 30 min on ice. After centrifugation (20,000 *g*, 10 min, 4° C), supernatant protein samples were separated by SDS-PAGE and transferred onto PVDF membranes (Bio-Rad Laboratories, CA), blocked with an Odyssey Blocking Buffer (IL-COR, Lincoln, NE). The membrane was incubated with the appropriate antibodies 1:600 (vol/vol) in the Odyssey Blocking Buffer with 0.2 % (vol/vol) Tween 20, followed by an incubation with a goat anti-rabbit IgG Alexa Fluor 680 secondary antibodies (Thermo Fisher Scientific, diluted at 3:10,000) in the Odyssey Blocking Buffer. The stained protein bands were digitally detected in an Odyssey Fc Dual-Mode Imaging System (IL-COR, Lincoln, NE).

### Statistical analyses

Each experiment was repeated at least three times. Results were expressed as mean ± standard deviation (SD). Data were assessed with one way ANOVA, followed by Student's t-test or Dunnet's multiple comparison test, and considered significantly different at *P *< 0.05.

## Results

### Effects of KLH on the NF-κB and/or AP-1 activities in THP-1 reporter cells

**Figure 1:**
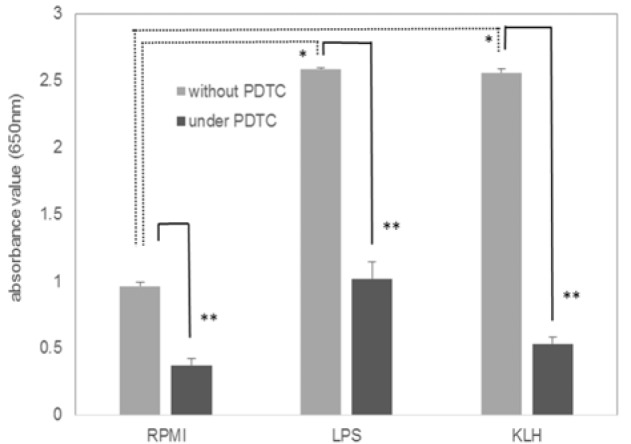
Effects of PDTC on the KLH-induced NF-κB activation. THP-1 reporter cell (THP1-XBlue™-MD2-CD14 Cells) was stimulated with 0.1 µg/ml LPS or 50 µg/ml KLH in the presence or the absence of 10 µM PDTC. Values represent mean ± SD of six assays. Significant differences from RPMI (negative control) values were defined as *P < 0.05 (one-way ANOVA and Dunnett's test), **P < 0.0001 (one-way ANOVA and Student's t-test).

NF-κB and/or AP-1 were slightly activated in the control THP1-XBlue™ -MD2-CD14 cells probably due to a serial culture passage of cells (Figure 1(Fig. 1)). The administration of LPS strongly activated NF-κB and/or AP-1 compared to the case in the control medium. An NF-κB inhibitor PDTC suppressed the activation induced by LPS. The administration of KLH markedly activated NF-κB and/or AP-1 as much as LPS, and PDTC also suppressed the KLH-induced activation.

### Syk inhibitor partially suppressed the KLH- induced NF-κB activation

**Figure 2:**
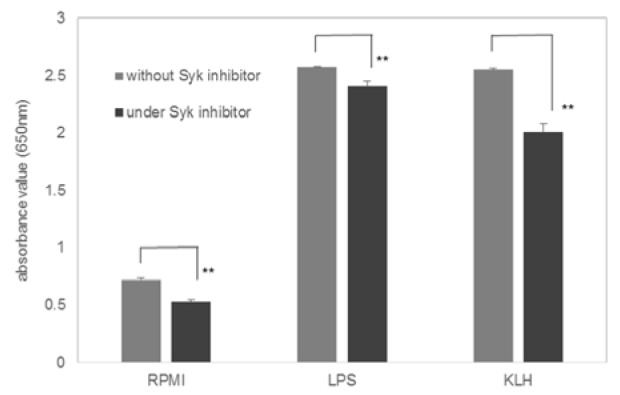
Effects of Syk inhibitor on the KLH-induced NF-κB activation. THP-1 reporter cell was stimulated with 0.1 µg/ml LPS or 50 µg/ml KLH in the presence or the absence of 1 µM Syk inhibitor (Bay 61-3606). Values represent mean ± SD of six assays. **Significantly different from values in the absence of the inhibitor, P < 0.0001 (one-way ANOVA and Student's t-test).

Syk is deeply involved in several innate immune receptors (Dennehy et al., 2008[13]; Robinson et al., 2009[43]; Sancho et al., 2009[45]; Kerrigan and Brown, 2010[23], 2011[24]). In THP1-XBlue™-MD2-CD14 cells, a Syk inhibitor, Bay 61-3606 hydrochloride, partially inhibited the KLH-induced NF-κB activation (Figure 2(Fig. 2)). 

### Erk inhibitor suppressed the KLH-induced NF-κB activation

**Figure 3:**
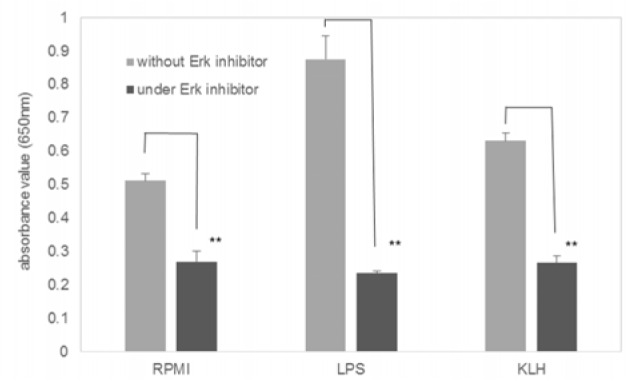
Effects of Erk inhibitor on the KLH-induced NF-κB activation. THP-1 reporter cell was stimulated with 0.1 µg/ml LPS or 50 µg/ml KLH in the presence or the absence of 5 µM Erk inhibitor (Nimbolide). Values represent mean ± SD of six assays. **Significantly different from values in the absence of the inhibitor, P < 0.0001 (one-way ANOVA and Student's t-test).

In THP1-Xblue™-MD2-CD14 cells, an Erk inhibitor, Nimble, obviously inhibited KLH-induced NF-κB activation (Figure 3(Fig. 3)). 

### Syk inhibitor partially suppressed the Erk phosphorylation in KLH-stimulated THP-1

Evaluated through Western blot with specific antibodies, anti-Erk1/2 antibody (EPR 17526) and anti-phospho-Erk1/2 (Tyr 202/204), it is suggested that Erk should be phosphorylated by KLH stimulation. The phosphorylation ratio significantly increased in a time-dependent manner. The peak of Erk phosphorylation was observed at 2 min after KLH stimulation and then the phosphorylated Erk decreased by 10 min after KLH stimulation. 

**Figure 4:**
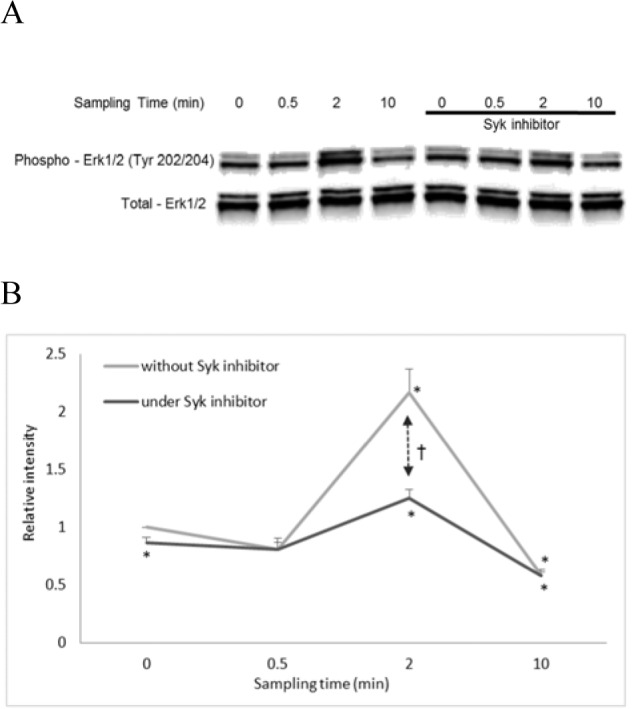
Effects of Syk inhibitor on the KLH-induced Erk phosphorylation. (A) THP-1 cells were pre-treated or non-treated 10 µM Syk inhibitor (Bay 61-3606) at 37° C for two hours. THP-1 cells were then stimulated with 500 µg/ml KLH at 37° C for various periods of time. At the end of the incubation period, cells were lysed in a RIPA buffer with inhibitors for 30 min on ice. After centrifugation (20,000 g, 10 min, 4° C), supernatant proteins were evaluated through the Western blot with antibodies, anti-Erk1/2 antibody (EPR 17526) and anti-phospho-Erk1/2 (Tyr 202/204). (B) Phosphorylation signals were normalized to total Erk1/2 signal intensities in the panel (A). The graphs represent mean ± SD of values obtained from three independent experiments. *Significantly different from the initial value in the absence of the inhibitor, *P < 0.05 (one-way ANOVA and Dunnett's test). †There is a significant difference between values with and without the inhibitor at 2 min, †P < 0.001 (one-way ANOVA and Student's t-test).

In contrast, in THP-1 cells treated with the Syk inhibitor, the phosphorylation ratio did not increase compared to the case of non-treated THP-1 cells, suggesting that the KLH-induced Erk phosphorylation was partially suppressed by the Syk inhibitor (Figure 4(Fig. 4)).

## Discussion

In this study, we have in part clarified the initial responses to KLH and direct effects of KLH on the innate immune systems in human monocytic cells. We have found that KLH activates NF-κB as much as LPS in the THP-1 cells and that the KLH-induced NF-κB activation is partially mediated via Syk and Erk pathways in human monocytic THP-1 cells. The NF-κB inhibitor, PDTC, suppresses the release of the inhibitory subunit IκB from the latent cytoplasmic form of NF-κB and does not influence the other DNA binding activities including AP-1 (Robinson et al., 2009[43]). PDTC markedly suppressed the KLH-induced activation of the NF-κB/AP-1 reporter cells, suggesting that the activation should be triggered by NF-κB. The transcription factor, NF-κB, plays crucial roles in the immune system (Ghosh et al., 1998[16]; Li and Verma, 2002[26]; Bonizzi and Karin, 2004[6]). We have then investigated signaling pathways from KLH reception to NF-κB activation in THP-1 monocyte. 

Presicce et al. (2008[40]) reported that KLH-induced activation and maturation of monocyte-derived dendritic cells (DCs) were partially mediated via one of lectin receptors, mannose receptor (MR). Mansour et al. (2006[30]) stated that mannose was important for MR-mediated endocytosis of monocyte-derived DCs. The KLH-induced NF-κB activation observed in this study might therefore have a certain association with a lectin receptor, probably because KLH was rich in mannose. The lectin receptors and other receptors, such as MR (Tsai et al., 2013[48]), CR3 (Xia et al., 1999[51]), TLRs (Netea et al., 2006[36]) and FcγRs (Vogelpoel et al., 2015[49]), drive Syk signaling cascades (Kerrigan et al., 2010[23]; Hadas et al., 2012[18]; Dennehy et al., 2008[13]; Falker et al., 2014[15]), leading to Syk autophosphorylation and NF-κB activation. The NF-κB activation results in the production of pro-inflammatory cytokines, which in turn induces cellular responses, including apoptosis and phagocytosis (Parsa et al., 2008[38]; Kingeter and Lin, 2012[25]; Falker et al., 2014[15]). In the present study, a Syk inhibitor, Bay 61-3606 hydrochloride, partially suppressed the KLH-induced NF-κB activation, suggesting that Syk would partially associate with the signaling pathways.

Erk has an important role of fundamental cellular reaction such as differentiation (Falker et al., 2014[15]), inflammatory responses (Roux and Blenis, 2004[44]; Zhang and Liu, 2002[52]) and apoptosis in mammalian cells (Parsa et al., 2008[38]; Roberts and Der, 2007[42]). Several studies reported that Erk was in downstream of these receptors, CR3(Li et al., 2014[27]), MR (Tsai et al., 2013[48]), TLR2 (Richardson et al., 2015[41]; Chen et al., 2015[8]) and FcγRs (Luo et al., 2010[28]; Song et al., 2004[46]). Many reports about phagocytosis mentioned that Erk had an important role in the phagocytosis of pathogenic organisms (Song et al., 2004[46]; Balagopal et al., 2006[3]; Parsa et al., 2008[38]) and that Syk promoted the phagocytosis of *F. tularensis* via Erk pathway (Parsa et al., 2008[38]). We demonstrated that an Erk inhibitor, Nimble, suppressed the KLH-induced NF-κB activation and that the KLH-induced Erk phosphorylation was partially suppressed by a Syk inhibitor. These results suggest that Erk would be located in downstream of Syk and upstream of NF-κB signaling pathways (Miyazaki et al., 2000[33]; Jiang et al., 2004[21]).

Syk and some Syk-related receptors are partially involved in the KLH-induced NF-κB activation and the KLH-induced Erk phosphorylation. It is necessary to identify KLH receptor(s) and the associated molecule(s) in order to understand the KLH-induced NF-κB activation mechanisms comprehensively. We are now further investigating the signaling cascades along these lines.

## References

[1] Amkraut AA, Malley A, Begley D (1969). Immunogenicity of hemocyanins and their subunits. J Immunol.

[2] Arancibia S, Espinoza C, Salazar F, Del Campo M, Tampe R, Zhong TY (2014). A novel immunomodulatory hemocyanin from the limpet Fissurella latimarginata promotes potent anti-tumor activity in melanoma. PLoS One.

[3] Balagopal A, MacFarlane AS, Mohapatra N, Soni S, Gunn JS, Schlesinger LS (2006). Characterization of the receptor-ligand pathways important for entry and survival of Francisella tularensis in human macrophages. Infect Immun.

[4] Becker MI, Arancibia S, Salazar F, Del Campo M, De Ioannes AE, Duc GHT (2014). Mollusk hemocyanins as natural immunostimulants in biomedical applications. Immune response activation.

[5] Ben Nasr A, Haithcoat J, Masterson JE, Gunn JS, Eaves-Pyles T, Klimpel GR (2006). Critical role for serum opsonins and complement receptors CR3 (CD11b/CD18) and CR4 (CD11c/CD18) in phagocytosis of Francisella tularensis by human dendritic cells (DC): uptake of Francisella leads to activation of immature DC and intracellular survival of the bacteria. J Leukoc Biol.

[6] Bonizzi G, Karin M (2004). The two NF-kappaB activation pathways and their role in innate and adaptive immunity. Trends Immunol.

[7] Butchar JP, Rajaram MV, Ganesan LP, Parsa KV, Clay CD, Schlesinger LS (2007). Francisella tularensis induces IL-23 production in human monocytes. J Immunol.

[8] Chen W-L, Sheu J-R, Chen R-J, Hsiao S-H, Hsiao C-J, Chou Y-C (2015). Mycobacterium tuberculosis Upregulates TNF-α Expression via TLR2/ERK signaling and induces MMP-1 and MMP-9 production in human pleural mesothelial cells. PLoS ONE.

[9] Clemens DL, Lee BY, Horwitz MA (2005). Francisella tularensis enters macrophages via a novel process involving pseudopod loops. Infect Immun.

[10] Curtis JE, Hersh EM, Butler WT, Rossen RD (1971). Antigen dose in the human immune response. Dose-relationships in the human immune response to Keyhole limpet hemocyanin. J Lab Clin Med.

[11] Curtis JE, Hersh EM, Freireich EJ (1970). Antigen-specific immunity in recipients of leukocyte transfusions from immune donors. Cancer Res.

[12] De Ioannes P, Moltedo B, Oliva H, Pacheco R, Faunes F, De Ioannes AE (2004). Hemocyanin of the molluscan Concholepas concholepas exhibits an unusual heterodecameric array of subunits. J Biol Chem.

[13] Dennehy KM, Ferwerda G, Faro-Trindade I, Pyż E, Willment JA, Taylor PR (2008). Syk kinase is required for collaborative cytokine production induced through Dectin-1 and Toll-like receptors. Eur J Immunol.

[14] Drummond RA, Saijo S, Iwakura Y, Brown GD (2011). The role of Syk/CARD9 coupled C-type lectins in antifungal immunity. Eur J Immunol.

[15] Falker K, Klarstrom-Engstrom K, Bengtsson T, Lindahl TL, Grenegard M (2014). The toll-like receptor 2/1 (TLR2/1) complex initiates human platelet activation via the src/Syk/LAT/PLCgamma2 signalling cascade. Cell Signal.

[16] Ghosh S, May MJ, Kopp EB (1998). NF-kappa B and Rel proteins: evolutionarily conserved mediators of immune responses. Annu Rev Immunol.

[17] Golovliov I, Kuoppa K, Sjöstedt A, Tärnvik A, Sandström G (1996). Cytokine expression in the liver of mice infected with a highly virulent strain of Francisella tularensis. FEMS Immunol Med Microbiol.

[18] Hadas S, Spira M, Hanisch UK, Reichert F, Rotshenker S (2012). Complement receptor-3 negatively regulates the phagocytosis of degenerated myelin through tyrosine kinase Syk and cofilin. J Neuroinflamm.

[19] Herscowitz HB, Harold WW, Stavitsky AB (1972). Immunochemical and immunogenic properties of a purified keyhole limpet haemocyanin. Immunology.

[20] Janeway CA, Medzhitov R (2002). Innate immune recognition. Annu Rev Immunol.

[21] Jiang B, Xu S, Hou X, Pimentel DR, Brecher P, Cohen RA (2004). Temporal control of NF-kappaB activation by ERK differentially regulates interleukin-1beta-induced gene expression. J Biol Chem.

[22] Katz J, Zhang P, Martin M, Vogel SN, Michalek SM (2006). Toll-like receptor 2 is required for inflammatory responses to Francisella tularensis LVS. Infect Immun.

[23] Kerrigan AM, Brown GD (2010). Syk-coupled C-type lectin receptors that mediate cellular activation via single tyrosine based activation motifs. Immunol Rev.

[24] Kerrigan AM, Brown GD (2011). Syk-coupled C-type lectins in immunity. Trends Immunol.

[25] Kingeter LM, Lin X (2012). C-type lectin receptor-induced NF-κB activation in innate immune and inflammatory responses. Cell Mol Immunol.

[26] Li Q, Verma IM (2002). NF-kappaB regulation in the immune system. Nat Rev Immunol.

[27] Li XJ, Goodwin CB, Nabinger SC, Richine BM, Yang Z, Hanenberg H (2014). Protein tyrosine phosphatase, Shp2, positively regulates macrophage oxidative burst. J Biol Chem.

[28] Luo Y, Pollard JW, Casadevall A (2010). Fcγ receptor cross-linking stimulates cell proliferation of macrophages via the ERK pathway. J Biol Chem.

[29] Malik M, Bakshi CS, Sahay B, Shah A, Lotz SA, Sellati TJ (2006). Toll-like receptor 2 is required for control of pulmonary infection with Francisella tularensis. Infect Immun.

[30] Mansour MK, Latz E, Levitz SM (2006). Cryptococcus neoformans glycoantigens are captured by multiple lectin receptors and presented by dendritic cells. J Immunol.

[31] Markl J, Savel-Niemann A, Wegener-Strake A, Süding M, Schneider A, Gebauer W (1991). The role of two distinct subunit types in the architecture of keyhole limpet hemocyanin (KLH). Naturwissenschaften.

[32] Medzhitov R, Janeway CA (1997). Innate immunity: the virtues of a nonclonal system of recognition. Cell.

[33] Miyazaki T, Katagiri H, Kanegae Y, Takayanagi H, Sawada Y, Yamamoto A (2000). Reciprocal role of ERK and NF-kappaB pathways in survival and activation of osteoclasts. J Cell Biol.

[34] Mogensen TH (2009). Pathogen recognition and inflammatory signaling in innate immune defenses. Clin Microbiol Rev.

[35] Moltedo B, Faunes F, Haussmann D, De Ioannes P, De Ioannes AE, Puente J (2006). Immunotherapeutic effect of Concholepas hemocyanin in the murine bladder cancer model: evidence for conserved antitumor properties among hemocyanins. J Urol.

[36] Netea MG, Gow NAR, Munro CA, Bates S, Collins C, Ferwerda G (2006). Immune sensing of Candida albicans requires cooperative recognition of mannans and glucans by lectin and Toll-like receptors. J Clinic Investig.

[37] Osorio F, Reis e Sousa C (2011). Myeloid C-type lectin receptors in pathogen recognition and host defense. Immunity.

[38] Parsa KV, Butchar JP, Rajaram MV, Cremer TJ, Tridandapani S (2008). The tyrosine kinase Syk promotes phagocytosis of Francisella through the activation of Erk. Mol Immunol.

[39] Parsa KV, Ganesan LP, Rajaram MV, Gavrilin MA, Balagopal A, Mohapatra NP (2006). Macrophage pro-inflammatory response to Francisella novicida infection is regulated by SHIP. PLoS Pathog.

[40] Presicce P, Taddeo A, Conti A, Villa ML, Della Bella S (2008). Keyhole limpet hemocyanin induces the activation and maturation of human dendritic cells through the involvement of mannose receptor. Mol Immunol.

[41] Richardson ET, Shukla S, Sweet DR, Wearsch PA, Tsichlis PN, Boom WH (2015). TLR2-dependent ERK signaling in Mycobacterium tuberculosis-infected macrophages drives anti-inflammatory responses and inhibits Th1 polarization of responding T cells. Infect Immun.

[42] Roberts PJ, Der CJ (2007). Targeting the Raf-MEK-ERK mitogen-activated protein kinase cascade for the treatment of cancer. Oncogene.

[43] Robinson MJ, Osorio F, Rosas M, Freitas RP, Schweighoffer E, Gross O (2009). Dectin-2 is a Syk-coupled pattern recognition receptor crucial for Th17 responses to fungal infection. J Exp Med.

[44] Roux PP, Blenis J (2004). ERK and p38 MAPK-activated protein kinases: a family of protein kinases with diverse biological functions. Microbiol Mol Biol Rev.

[45] Sancho D, Joffre OP, Keller AM, Rogers NC, Martínez D, Hernanz-Falcón P (2009). Identification of a dendritic cell receptor that couples sensing of necrosis to immunity. Nature.

[46] Song X, Tanaka S, Cox D, Lee SC (2004). Fcgamma receptor signaling in primary human microglia: differential roles of PI-3K and Ras/ERK MAPK pathways in phagocytosis and chemokine induction. J Leukoc Biol.

[47] Stenmark S, Sunnemark D, Bucht A, Sjöstedt A (1999). Rapid local expression of interleukin-12, tumor necrosis factor alpha, and gamma interferon after cutaneous Francisella tularensis infection in tularemia-immune mice. Infect Immun.

[48] Tsai YM, Hsu SC, Zhang J, Zhou YF, Plunkett B, Huang SK (2013). Functional interaction of cockroach allergens and mannose receptor (CD206) in human circulating fibrocytes. PLoS One.

[49] Vogelpoel LTC, Baeten DLP, de Jong EC, den Dunnen J (2015). Control of cytokine production by human fc gamma receptors: implications for pathogen defense and autoimmunity. Front Immunol.

[50] Weigle WO (1964). Immunochemical properties of hemocyanin. Immunochemistry.

[51] Xia Y, Vetvicka V, Yan J, Hanikyrova M, Mayadas T, Ross GD (1999). The beta-glucan-binding lectin site of mouse CR3 (CD11b/CD18) and its function in generating a primed state of the receptor that mediates cytotoxic activation in response to iC3b-opsonized target cells. J Immunol.

[52] Zhang W, Liu HT (2002). MAPK signal pathways in the regulation of cell proliferation in mammalian cells. Cell Res.

